# Stress management by benzodiazepines in septic shock and critical illness: mechanistic and clinical evidence supporting alternative sedative strategies

**DOI:** 10.15698/cst2026.08.320

**Published:** 2026-08-07

**Authors:** Flavia Lambertucci, David Skurnik, Damien Roux, Laurence Arbibe, Lucillia Bezu, Isabelle Martins, Guido Kroemer

**Affiliations:** 1 Team “Metabolism, Cancer & Immunity”, Équipe Labellisée par la Ligue Contre le Cancer, Centre de Recherche des Cordeliers, Inserm UMRS1138, Université Paris Cité, Sorbonne Université, Paris, France; 2 Université Paris Cité, INSERM, CNRS, Institut Necker Enfants Malades, Paris, France; 3 Service de Microbiologie Clinique, AP-HP, Hôpital Necker Enfants Malades, Paris, France; 4 AP-HP, Hôpital Louis Mourier, DMU ESPRIT, Service de Médecine Intensive Réanimation, Colombes, France; 5 Université Paris Cité, INSERM UMR-S1151, CNRS UMR-S8253, Institut Necker Enfants Malades, Paris, France; 6 Université Paris Cité, INSERM, CNRS, Institut Necker Enfants Malades, Paris, France; 7 Metabolomics and Cell Biology Platforms, Institut Gustave Roussy, Villejuif, France; 8 Intensive Care Unit, Gustave Roussy, Villejuif, France; 9 Institut du Cancer Paris CARPEM, Department of Biology, Hôpital Européen Georges Pompidou, AP-HP, Paris, France

**Keywords:** acyl-CoA binding protein, bacterial clearance, diazepam binding inhibitor, emergency medicine, infection, intensive care

## Abstract

Critical care involves the management of organismal stress by sedation. Benzodiazepines remain widely used for anxiolysis and sedation in critical care, including in septic shock. Yet converging mechanistic, preclinical and clinical evidence suggests that benzodiazepines and the endogenous ligand of benzodiazepine-binding sites, the “endozepine” acyl-CoA binding protein/diazepam binding inhibitor (ACBP/DBI), may impair host immune defenses, exacerbate organ dysfunction and contribute to long-term morbidity after intensive care. A unifying framework is that benzodiazepines may accelerate “iatrogenic aging” by antagonizing adaptive stress responses (notably autophagy) and favoring cellular senescence, chronic inflammation, immunosuppression and neurocognitive perturbations. Here, we synthesize evidence supporting benzodiazepine avoidance in sepsis-heavy intensive care populations. We propose that replacing benzodiazepines with alternative sedative strategies may improve both short-term outcomes and long-term trajectories of post-sepsis disability. We hypothesize that post-intensive-care syndrome (PICS), including -as a specific case- post-sepsis syndrome (PSS), is caused by premature aging of the organism and that PICS/PSS might be attenuated by the avoidance of benzodiazepine administration during and after critical care.

## INTRODUCTION

Benzodiazepines are among the most frequently used psychoactive drugs worldwide and remain common in ICU sedation regimens, particularly where rapid anxiolysis, anticonvulsant activity or deep sedation is required 1, 2. However, benzodiazepines are increasingly viewed as biologically active modifiers of immune-inflammatory, metabolic and neurocognitive pathways -not merely symptomatic therapies 1–5. This perspective is especially relevant in septic shock and critical illness, where survival depends on calibrated inflammation, effective antimicrobial defense, metabolic flexibility, and preservation of brain function.

**Figure 1:**
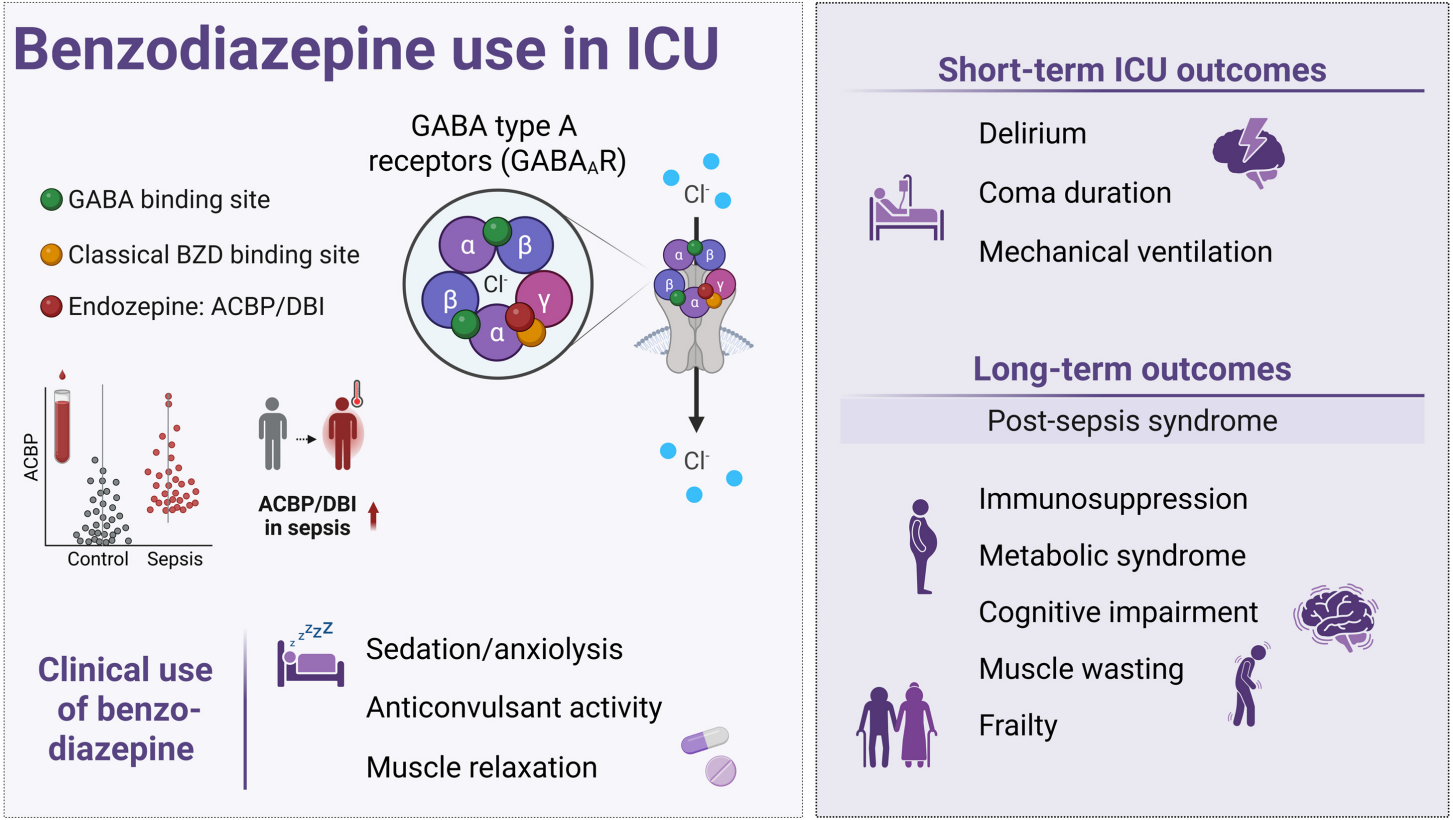
Benzodiazepine (BZD) use in the intensive care unit (ICU): mechanisms and clinical outcomes. BZDs act on the γ -aminobutyric acid (GABA) type A receptor (GABA type A receptor), by binding to a site distinct from the GABA binding site. Endozepines such as acyl-CoA–binding protein/diazepam-binding inhibitor (ACBP/DBI) are increased in sepsis. In the ICU, BZDs are used for sedation/anxiolysis, anticonvulsant therapy, and muscle relaxation. Associated outcomes include delirium, prolonged coma and mechanical ventilation, and long-term sequelae consistent with post-sepsis syndrome, including immunosuppression, metabolic dysregulation, cognitive impairment, muscle wasting, and frailty.

The concept of “iatrogenic aging” posits that certain medications can accelerate hallmarks of biological aging, thereby worsening age-associated pathologies and reducing resilience to stress 6. A mechanistic entry point is the tissue stress hormone acyl-CoA binding protein/diazepam binding inhibitor (ACBP/DBI). ACBP/DBI is also termed “endozepine” (a neologism referring to its role as the endogenous equivalent of benzodiazepines) because it competes with the prototypic benzodiazepine diazepam for binding to gamma-aminobutyric acid (GABA) type receptors (GABA ${}_{A}$ R) 7–9. As diazepam, ACBP/DBI acts as a positive allosteric modulator on GABA ${}_{A}$ R, which is broadly expressed, not only in the central nervous system, but also in multiple different peripheral cell types including epithelial cells (e.g. hepatocytes) and myeloid cells 10–13 (Figure 1).

ACBP/DBI has emerged as a targetable autophagy checkpoint and a mediator of pathological aging trajectories. In humans, plasma ACBP/DBI concentrations correlate with chronological age and cardiometabolic risk and predict future cardiovascular events and cancer diagnoses independent of age and body mass index (BMI), supporting its role as a biomarker of “biological” aging 14–16. In centenarians, elevated ACBP/DBI associates with deterioration, reduced glomerular filtration, and increased senescence-associated cytokines 17. Experimentally, antibody-mediated ACBP/DBI neutralization reduces aging-like phenotypes (including senescence-linked readouts) in diverse contexts, consistent with systemic geroprotective activity 17, 18.

Benzodiazepine treatment affects the clinical outcome of septic shock at two temporal levels, short term and long term. Acutely, benzodiazepine-centered sedation (often using midazolam or, less commonly, lorazepam) is associated with more delirium/brain dysfunction and longer mechanical ventilation compared with dexmedetomidine or propofol-based approaches. In randomized clinical trials (RCT) performed in intensive care units (ICU), dexmedetomidine reduced delirium and ventilation duration versus midazolam 19, 20. Moreover, in sepsis-focused analyses, dexmedetomidine improved delirium/coma-free days, ventilator-free days and mortality as compared to lorazepam 21. Chronically, many sepsis survivors develop post-intensive-care syndrome (PICS) and post-sepsis syndrome (PSS), which include accelerated muscle wasting 22, cognitive and psychiatric dysfunction 23, 24, chronic kidney disease progressing to end-stage renal disease 25, sustained immunosuppression with recurrent infections 26, 27, and excess cardiovascular events 28, 29. Collectively, this resembles an accelerated aging phenotype, raising the hypothesis that sedative exposure and endozepine biology might influence not only ICU outcomes, but also long-term trajectories of disability and senescence-linked organ decline.

In this review, we advance the hypothesis that benzodiazepines should be preferentially substituted by alternative sedative strategies in septic shock and critical illness, and that ACBP/DBI is a mechanistically grounded biomarker and therapeutic target linking sedation biology, immune dysfunction and accelerated aging.

## PRECLINICAL EVIDENCE

### Benzodiazepines impair antimicrobial defense in preclinical studies

**Figure 2:**
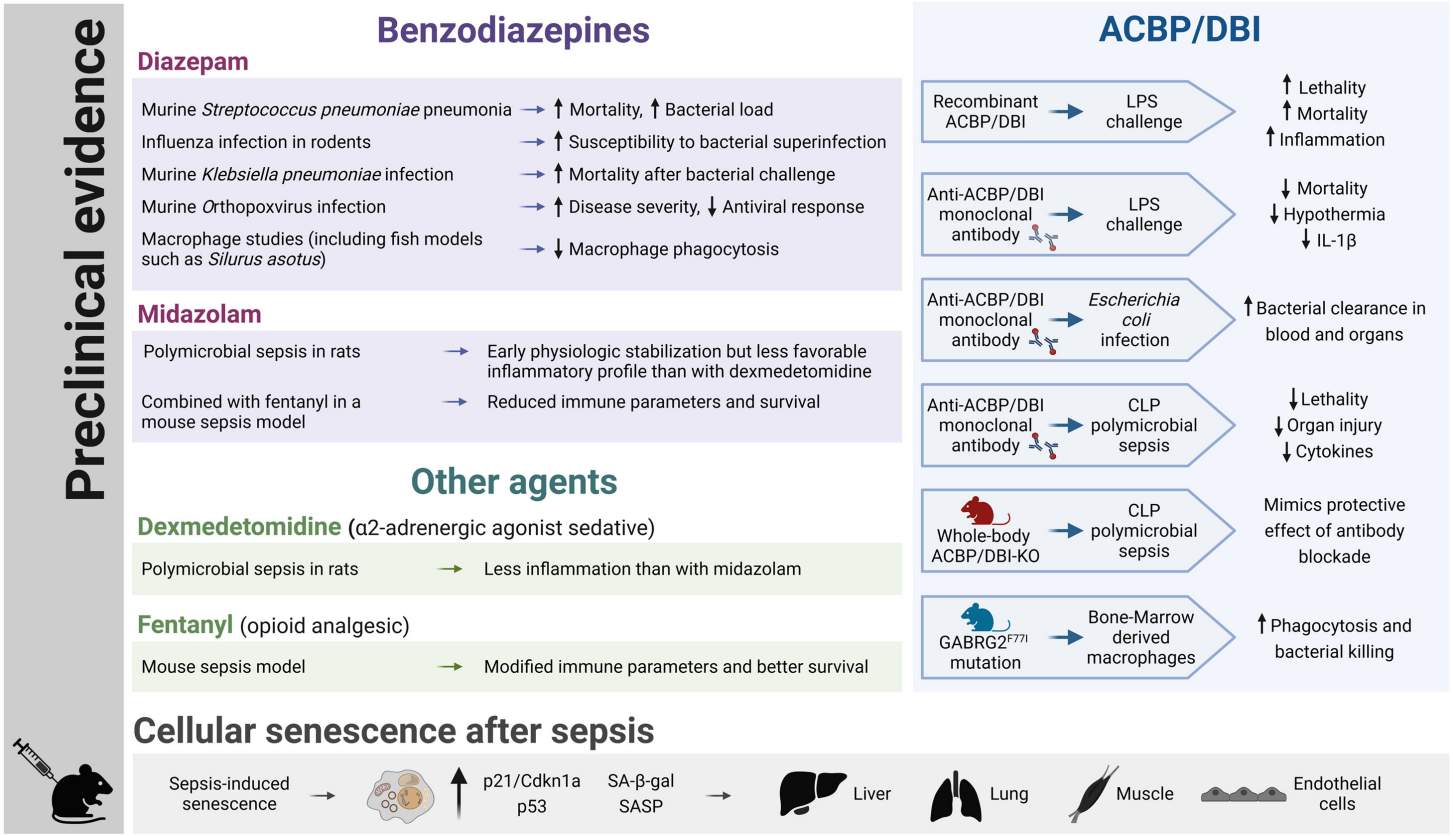
Preclinical evidence for benzodiazepines in sepsis. BDZs (e.g., diazepam, midazolam) are associated with increased mortality, impaired bacterial clearance, and dysregulated immune responses in sepsis models. Other agents, like dexmedetomidine shows reduced inflammation comparted to BZDs, while fentanyl may improve survival. In preclinical models, ACBP/DBI increase mortality and inflammation, with its neutralization improving bacterial clearance and survival. Sepsis-induced cellular senescence contributes to multiorgan dysfunction.

Complementing emerging translational sepsis studies, multiple animal models indicate that benzodiazepine exposure can increase susceptibility to infection and worsen outcomes. Diazepam increased mortality and bacterial load in murine *Streptococcus pneumoniae* pneumonia; these effects were reversed by the GABA ${}_{A}$ R antagonist bicuculline, supporting a receptor-dependent immunosuppressive mechanism 30. In rodent influenza models, diazepam exposure increased vulnerability to bacterial superinfection and highlighted dynamic regulation of GABA ${}_{A}$ R subunits on immune cells, including alveolar macrophages and blood monocytes, providing a mechanistic substrate for sedation–immunity crosstalk 31. Accordingly, diazepam inhibits the phagocytic activity of macrophages in other vertebrate species including catfish (*Silurus asotus*) 32. Earlier work reported that diazepam pretreatment increased mortality in mice challenged with *Klebsiella pneumoniae*, consistent with impaired natural resistance 33. In viral infection, diazepam enhanced severity of orthopoxvirus disease in mice and reduced antiviral immune responses, suggesting that benzodiazepines can compromise protective immunity beyond bacteria 34. Although the comparison of drug doses among different species is inherently challenging, the benzodiazepines doses used in these studies, when adjusted using standard allometric scaling 35, fall within or close to clinically relevant exposure ranges (Figure 2).

Not all sepsis-related rodent sedation studies show uniform harm at all timepoints; for example, sedation itself may improve early physiologic stability in severe sepsis models, and comparative effects can evolve over time. In polymicrobial severely septic rats, both midazolam and dexmedetomidine sedation improved early outcome, whereas dexmedetomidine showed more favorable effects on interleukin (IL)-6 and apoptosis over a prolonged course, supporting the idea that sedative choice may influence downstream immunopathology and recovery trajectories 36. Moreover, in a murine sepsis model, fentanyl and/or midazolam altered immune parameters and survival, underscoring that sedative-analgesic combinations can modulate sepsis biology rather than simply providing comfort measures 37. Taken together, these studies support biological plausibility: benzodiazepines can act as immunomodulators that -depending on context, timing, and comparator- may impair pathogen clearance or skew immune responses toward maladaptive profiles.

### Endozepine (ACBP/DBI) as a driver of septic shock pathogenesis

We recently demonstrated that mice exhibit increased plasma ACBP/DBI in three distinct sepsis models (1. endotoxemia induced by injection of bacterial lipopolysaccharide [LPS], 2. Live *Escherichia coli* infection, and 3. polymycrobial sepsis induced by cecal ligation and puncture [CLP]), strongly suggesting that microbes and microbial products may elicit an evolutionarily conserved surge of circulating endozepines 38.

In the same study, prophylactic anti-ACBP/DBI monoclonal antibody (mAb) administration reduced mortality following LPS challenge, blunted hypothermia, and reduced IL-1 β elevation. LPS-induced cardiac injury (troponin) was reduced (trend), and echocardiography demonstrated partial prevention of LPS-associated cardiac dysfunction, with improvements in heart rate and left ventricular ejection fraction. Conversely, recombinant ACBP/DBI -nontoxic alone- heightened LPS lethality, directly supporting a pathogenic (driver) role for ACBP/DBI in septic shock 38.

Pretreatment with anti-ACBP/DBI mAb strongly reduced lethality after intraperitoneal *E. coli* inoculation and improved bacterial clearance in blood, peritoneal lavage, spleen, and kidney, with reduced AST elevations. Depleting macrophages or neutrophils compromised the clearance-enhancing effect, implicating myeloid effectors. Therapeutically, administration after infection reduced splenic bacterial burden. *In vitro*, anti-ACBP/DBI enhanced neutrophil internalization and killing of *E. coli*, and improved phagocytosis/killing by alternatively activated macrophages, suggesting phenotype-specific immunomodulation. Notably, macrophages from Gabrg2 ${}^{\mathrm{F77I/F77I}}$ mice (a loss-of-function mutation abolishing the binding of ACBP/DBI and benzodiazepines to GABA ${}_{A}$ R) exhibited constitutively increased phagocytosis and killing, supporting the idea that endozepine signaling constrains antibacterial effector functions 38.

In CLP, prophylactic anti-ACBP/DBI mAb delayed lethality, blunted hypothermia, improved renal function (reduced blood urea nitrogen), reduced hepatic transaminases, reduced circulating ACBP/DBI (partially), and diminished multiple inflammatory cytokines. High-dimensional immune profiling revealed a shift away from CLP-induced pro-inflammatory myeloid subpopulations and expansion of CD206 ${}^{+}$ anti-inflammatory subsets across organs (kidney, heart, spleen, liver), consistent with myeloid landscape remodeling. Critically, ACBP/DBI neutralization reduced bacterial burden in peritoneal fluid and internal organs, demonstrating improved microbial clearance in polymicrobial sepsis 38. Liver bulk transcriptomics showed CLP-induced inflammatory gene programs that were downregulated by ACBP/DBI blockade and identified CLP-driven upregulation of the pro-senescence gene *Cdkn1a* (which encodes the protein p21, a prototypic marker of cellular senescence) that was blunted by ACBP/DBI inhibition; immunohistochemistry confirmed reduced hepatic p21 ${}^{+}$ cells after ACBP/DBI neutralization. Lung transcriptomics and histology indicated attenuation of CLP-driven gene induction and reduced granulocytic infiltration. Mechanistic specificity was reinforced by three findings: (i) diazepam abolished the survival benefit of anti-ACBP/DBI mAb; (ii) whole-body Dbi knockout mimicked mAb effects; and (iii) anti-ACBP/DBI improved survival even when administered 6 hours after CLP, supporting therapeutic potential after microbial translocation and after ACBP/DBI surge 38.

### Cellular senescence after septic shock

Cellular senescence is a stable stress response characterized by durable cell-cycle arrest (usually through the upregulation of cyclin-dependent kinase inhibitors including CDKN1A/p21), metabolic rewiring, mitochondrial dysfunction, chromatin remodeling and a pro-inflammatory senescence-associated secretory phenotype (SASP) 39–41. While senescence can limit malignant transformation and participate in tissue remodeling, persistent or widespread senescence contributes to organ dysfunction, chronic inflammation, frailty, and immunosenescence—features that mirror PICS/PSS.

Recent preclinical work directly supports senescence activation after sepsis and ICU-relevant injury: in mice, CLP polymicrobial sepsis triggered rapid hepatic senescence within 24 hours, increasing p21/Cdkn1a, p53, senescence-associated β -galactosidase, and SASP transcripts across multiple hepatic cell types; senolytic treatment (dasatinib + quercetin) reduced mortality 42. CLP also induced early pulmonary stress-senescence signaling with increased p21 and p21 ${}^{+}$ cells in lung during sepsis-associated acute lung injury 43. In skeletal muscle, CLP or LPS provoked weakness with senescence features; metformin attenuated senescence markers and improved strength 44. In rats, septic shock induced stress-induced premature senescence in conduit vessels with persistent endothelial dysfunction, providing a mechanistic hypothesis for post-sepsis cardiovascular risk 45. Thus, the current state of literature on rodent models describes sepsis-induced senescence in multiple cell types in the liver, lung, skeletal muscle and endothelial cells. In humans, integrated transcriptomics and scRNA-seq reanalysis performed on circulating leukocytes demonstrated enrichment of senescence-associated gene modules in immune compartments during sepsis, supporting activation of senescence-linked programs clinically 46. Importantly, this concept is now being translated into interventional studies, as exemplified by the ongoing STOP-Sepsis trial 47, a multicenter, randomized, double-blinded study evaluating the senolytic agent fisetin in elderly septic patients, which will directly test whether pharmacological clearance of senescent cells can mitigate organ dysfunction and improve outcomes.

Our recent work adds a particularly important node to this network: in CLP, hepatic *Cdkn1a* (p21) induction and p21 ${}^{+}$ cell accumulation were blunted by anti-ACBP/DBI mAb, linking endozepine biology directly to a sepsis-induced senescence marker program 38. This aligns with independent evidence that ACBP/DBI is a driver of pathological aging and that its neutralization can prevent senescence-like organ decline in other injury contexts 17, 18. Collectively, these findings support a model in which sepsis can trigger an accelerated aging phenotype, and that ACBP/DBI -acting as an endogenous benzodiazepine-like signal- may contribute to senescence induction, immune dysfunction, and impaired long-term recovery.

In summary, ACBP/DBI rises as a conserved stress signal and functions as a driver of maladaptive inflammation, multiorgan injury and impaired bacterial clearance; these outcomes are reversible by ACBP/DBI neutralization or *Dbi* gene deletion and the resulting protective effects are abolished by co-administration of diazepam. In parallel, sepsis activates senescence programs that can be pharmacologically modulated. We therefore hypothesize that sepsis/critical illness can accelerate biological aging, and that ACBP/DBI and benzodiazepine signaling are plausible mediators, motivating biomarker-guided clinical studies and interventional trials.

## CLINICAL EVIDENCE

### ACBP/DBI as a prognostic biomarker in human sepsis and septic shock

**Figure 3:**
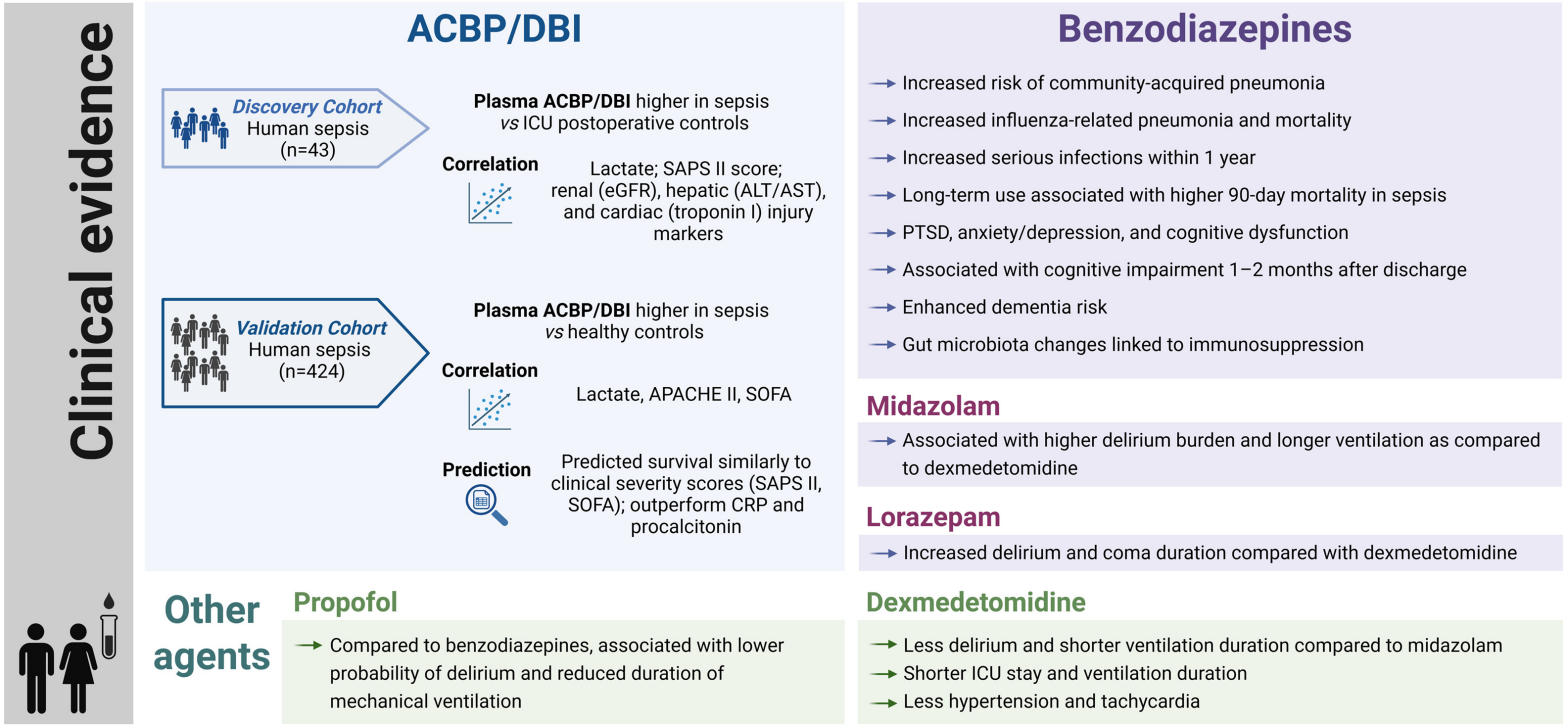
Clinical evidence for benzodiazepines and related agents in critical illness. In sepsis cohorts, elevated ACBP/DBI levels correlate with disease severity and adverse outcomes, while also predicting worse clinical trajectories. Benzodiazepines (midazolam, lorazepam) are associated with increased delirium, prolonged coma, and worse long-term outcomes compared with alternative sedatives. In contrast, dexmedetomidine and propofol are associated with reduced delirium and shorter ventilation duration.

Recently, we found that, in a discovery human cohort (n=43), plasma ACBP/DBI increased in septic shock compared to ICU postoperative aseptic inflammation controls, and it was higher in non-survivors than survivors 38. ACBP/DBI correlated with lactate, supporting alignment with hypoperfusion, stress metabolism, and prognosis. ACBP/DBI also correlated with biochemical markers of renal (lower estimated glomerular filtration rate), hepatic (alanine and aspartate aminotransferases), and cardiac injury (troponin I), supporting a relationship with multiorgan injury. Importantly, ACBP/DBI predicted survival with performance comparable to a clinical score (SAPS II) that is widely used in ICU patients to estimate hospital mortality risk and prognosis of patients with sepsis. These findings were replicated in a large validation cohort (n = 424): ACBP/DBI was increased in sepsis and numerically higher in septic shock versus controls (healthy and noninfectious SIRS -systemic inflammatory response syndrome- patients). Levels were higher in sepsis non-survivors (and trended higher in septic shock non-survivors), correlated with lactate, clinical scores (APACHE II, Acute Physiology and Chronic Health Evaluation II, and SOFA, Sequential Organ Failure Assessment) 48, 49, and outperformed C reactive protein (CRP) and procalcitonin for distinguishing survivors from non-survivors, with receiver operating curves (ROC) areas under the curve (AUC) comparable to SOFA and APACHE II. Kaplan–Meier stratification by median ACBP/DBI showed worse prognosis for patients with higher levels 38 (Figure 3).

Conceptually, these data position ACBP/DBI alongside (or integrated into) clinical scoring systems: it is mechanistically anchored, rises across species after microbial challenge, and predicts death similarly to multi-parameter physiologic scores, suggesting it captures core biology rather than merely reflecting one organ axis. Prior work also supports that endozepines rise in systemic inflammation in patients 50–52, lending independent plausibility.

### Benzodiazepines effects, severe infection and immunosuppression

Population-based studies associate benzodiazepines to infection risk in ways that plausibly influence sepsis onset, severity, and post-ICU vulnerability. Benzodiazepine exposure has been associated with increased risk of community-acquired pneumonia and worse short- and long-term outcomes among those with pneumonia 53. In UK primary care data, benzodiazepines were associated with increased likelihood of influenza-like illness–related pneumonia and mortality, supporting infection susceptibility signals outside the ICU-sedation setting 54. More recently, a large Swedish register-based study triangulating multiple designs (matched cohort, co-twin control, active comparator) found that incident benzodiazepine and Z-drug (BZDR) initiation was associated with increased risk of serious infections (viral and bacterial) within one year, with a dose-response relationship across cumulative exposure categories 55. This is notable because the study attempted to attenuate confounding by indication using active comparators and familial controls, leaving a residual signal consistent with immune modulation. A retrospective population-based cohort study in South Korea reported that long-term benzodiazepine use was associated with increased 90-day mortality among adult patients with sepsis compared with non-users 56.

While residual confounding remains possible (psychiatric comorbidity, frailty, baseline function, medication burden), these findings align with the biological plausibility established by our rodent infection models and with our mechanistic observation that diazepam can reverse the benefit of blocking endogenous endozepine signaling in polymicrobial sepsis in mice 38. Moreover, the human studies suggesting immunosuppressive effects of benzodiazepines in the context of infectious disease (see above) and cancer 4, 57, 58 are compatible with results in mice, showing that endozepine neutralization has broad immunostimulatory effects 16, 59, 60. It is noteworthy that the use of benzodiazepines in patients is associated with long-lasting shifts in the intestinal microbiota towards an immunosuppression-associated composition 58, 61. Whether such shifts are the cause or consequence of benzodiazepine-induced immunosuppression remains to be determined.

### ICU sedation with benzodiazepines compared to other agents

A broad literature supports minimizing benzodiazepines during ICU sedation to reduce delirium and shorten ventilation. In a very large national cohort of mechanically ventilated ICU patients (MV ≥ 48 h), Lee *et al*. reported that non-benzodiazepine exposure was associated with lower in-hospital mortality compared with benzodiazepine use, though confounding by indication remains a central limitation (depth of sedation, severity, neurologic injury, etc.) 62. In a propensity-matched cohort analysis comparing sedatives among critically ill patients (including acute respiratory distress syndrome [ARDS]), dexmedetomidine exposure was associated with lower in-hospital mortality than midazolam and propofol in the reported comparisons, while all sedated groups had longer stays vs no sedation, again highlighting the difficulty of separating agent effects from sedation strategy and illness phenotype 63.

Several randomized clinical trials (RCTs) have compared the use of benzodiazepines, in particular midazolam and lorazepam, with alternative sedatives such as the alpha-2 adrenergic agonist dexmedetomidine. Across RCTs, prespecified subgroup analyses, and large observational datasets, benzodiazepine-based ICU sedation was repeatedly associated with more delirium/brain dysfunction, longer ventilation, compared with dexmedetomidine -or propofol-forward strategies- particularly relevant in sepsis where baseline delirium risk and encephalopathy are common. In the large multicenter RCT SEDCOM enrolling mechanically ventilated ICU patients, dexmedetomidine and midazolam achieved similar time in target sedation, but dexmedetomidine was associated with less delirium and shorter ventilation; bradycardia was the key adverse effect signal 19. The PRODEX/MIDEX program led to the discovery that in patients requiring prolonged mechanical ventilation, dexmedetomidine was not inferior for maintaining light-to-moderate sedation and was associated with reduced ventilation duration vs midazolam, with differences in adverse-effect profiles (notably hemodynamic effects) 20. In the SPICE III trial enrolling 3,904 mechanically ventilated ICU patients, early dexmedetomidine-based sedation did not affect overall 90-day mortality compared with usual-care sedation. However, a prespecified subgroup analysis suggested age-dependent heterogeneity, with a higher probability of death among younger patients, particularly non-operative patients below the cohort median age of 63.7 years, and a possible benefit among older patients 64, 65. A sepsis-specific meta-analysis of RCTs found no statistically significant reduction in all-cause mortality with dexmedetomidine, but did find associations with shorter ICU/hospital stay and shorter ventilation duration, alongside increased bradycardia risk, supporting the view that benefits are often in “ICU efficiency” and delirium-related domains rather than consistent mortality reduction 66.

Together, these data support a pragmatic clinical stance: even where mortality effects are heterogeneous, the consistent reduction in delirium burden and ventilation duration is highly relevant in septic shock -where encephalopathy is frequent and where prolonged ventilation increases infection risk, immobility and downstream PICS.

PICS includes neuropsychiatric morbidity, and benzodiazepines have been implicated as risk factors for post-traumatic stress disorder, anxiety/depression, and cognitive dysfunction in ICU survivors 67, 68. In sepsis survivors specifically, cognitive dysfunction 1-2 months after discharge has been reported to be increased in patients who received benzodiazepines, with benzodiazepine use emerging as an independent factor alongside severity indices in the described analysis 69. Observational signals also link benzodiazepine exposure to dementia risk, though confounding by prodromal symptoms and reverse causality remain important interpretive constraints 70.

The translational implication is that benzodiazepines may not only worsen acute delirium but could also intersect with senescence and aging pathways, especially if they amplify endozepine-like signaling that suppresses autophagy and promotes inflammatory and senescence-associated cytokine milieus 17, 18. Making this mechanistic link explicit strengthens the rationale for avoiding benzodiazepine use during and after critical illness. This hypothesis is testable by means of a tripartite strategy: (i) incorporate ACBP/DBI measurements into sedation strategy trials, (ii) evaluate senescence biomarkers longitudinally and (iii) track PICS endpoints.

## CONCLUSION AND OUTLOOK

Septic shock is a potentially lethal syndrome defined by dysregulated host responses leading to multiorgan failure. Survivors frequently experience long-term disability, frailty, immune dysfunction and cardiometabolic decline that collectively resemble accelerated aging. An emerging mechanistic thread is cellular senescence, which is induced during critical illness in multiple organs and immune compartments, and can be modified by senolytic or metabolic interventions.

Within this landscape, benzodiazepines occupy a precarious position. Clinically, benzodiazepine-centered ICU sedation is repeatedly associated with greater delirium and longer ventilation than dexmedetomidine-forward approaches in general ICU populations and in sepsis-focused analyses. Population data also link benzodiazepines to pneumonia and serious infections and to increased mortality in sepsis with long-term pre-exposure. Preclinically, benzodiazepines can worsen bacterial and viral infection outcomes in rodents, often through benzodiazepine-sensitive GABA ${}_{A}$ R signaling on immune cells. Moreover, elevation of the endogenous benzodiazepine equivalent or “endozepine” ACBP/DBI in the plasma indicates poor prognosis in patients with septic shock. In mice, ACBP/DBI mediated broad pro-inflammatory and pro-senescence effects. Across murine models (LPS, *E. coli*, CLP), ACBP/DBI neutralization improved survival, enhanced bacterial clearance via myeloid effectors, mitigated multiorgan injury, remodeled myeloid landscapes, and crucially, blunted a hepatic senescence marker program. Diazepam reversed the benefit of ACBP/DBI neutralization in CLP, providing experimental evidence that benzodiazepine-like signaling can worsen septic shock pathogenicity.

Taken together, decades of clinical observations, preclinical data, and our mechanistic study converge to a clear message: benzodiazepine use in septic shock is associated with predictable and presumably preventable risks. The available evidence suggests that clinicians handling ICU patients should avoid benzodiazepines whenever feasible, prioritize light sedation, and favor dexmedetomidine or propofol centered regimens with protocolized titration, delirium prevention bundles and early mobilization. Accordingly, the Society of Critical Care Medicine (SCCM, United States) and the European Society of Intensive Care Medicine (ESICM) endorse the PADIS (Pain, Agitation/Sedation, Delirium, Immobility and Sleep disruption) guideline that contains a low-quality evidence-based recommendation for minimizing benzodiazepine use for routine ICU sedation outside of legitimate indications such as status epilepticus, alcohol or benzodiazepine withdrawal syndromes or counterindications of alternative sedatives such as dexmedetomidine and propofol 71. In our opinion, the quality of evidence supporting this recommendation should be updated to a higher level.

In ICU patients, minimization or total suppression of benzodiazepine use can be expected to consistently reduce ventilation duration, the risk of delirium burden, as well as long-term neuro-cognitive effects. In addition, it may be interesting to evaluate the endozepine ACBP/DBI as a stratification biomarker and therapeutic target in ICU patients. Near-term studies should test whether ACBP/DBI levels predict who benefits most from benzodiazepine avoidance, whether benzodiazepines acutely mimic the increased circulating effects of ACBP/DBI in critical illness, and whether ACBP/DBI trajectories correlate with senescence biomarkers and PICS endpoints. Ultimately, interventional trials of ACBP/DBI antagonism (antibodies or small molecules) could assess whether blocking endozepine signaling improves survival, reduces organ dysfunction and attenuates post-sepsis accelerated aging.

In sum, the traditional view of benzodiazepines as interchangeable sedatives is increasingly untenable in septic shock. Our study suggests that the risks of benzodiazepines outweigh their benefits and supports a precision ICU sedation strategy that pairs benzodiazepine minimization with ACBP/DBI-informed interventions to improve both short-term survival and long-term resilience after critical illness.

## AUTHOR CONTRIBUTIONS

**G.K.** wrote the main manuscript with the input of all other authors. **F.L.** prepared the figures with the help of **I.M.** All authors reviewed the manuscript.

## CONFLICT OF INTEREST

FL, IM and GK are co-inventors of a patent application describing the therapeutic of ACBP/DBI neuralization in sepsis. IM is a co-founder of Sanvitalia and has been consulting for Osasuna Therapeutics. GK has been holding research contracts with Daiichi Sankyo, Eleor, Kaleido, Lytix Pharma, PharmaMar, Osasuna Therapeutics, Samsara Therapeutics, Sanofi, Sutro, Tollys, and Vascage. GK is on the Board of Directors of the Bristol Myers Squibb Foundation France. GK is a scientific co-founder of everImmune, Osasuna Therapeutics, Samsara Therapeutics, Sanvitalia and Therafast Bio. GK is in the scientific advisory boards of Hevolution, Institut Servier, and Rejuveron Life Sciences/Centenara Labs AG. GK is the inventor of patents covering therapeutic targeting of aging, cancer, cystic fibrosis and metabolic disorders. GK’s brother, Romano Kroemer, was an employee of Sanofi and now consults for Boehringer-Ingelheim. GK’s wife, Laurence Zitvogel, has held research contracts with Glaxo Smyth Kline, Incyte, Lytix, Kaleido, Innovate Pharma, Daiichi Sankyo, Pilege, Merus, Transgene, 9 m, Tusk and Roche, was on the on the Board of Directors of Transgene, is a cofounder of everImmune, and holds patents covering the treatment of cancer and the therapeutic manipulation of the microbiota. The funders had no role in the design of the study; in the writing of the manuscript, or in the decision to publish the results.

## ABBREVIATIONS

ACBP – acyl-CoA-binding protein

APACHE II – acute physiology and chronic health evaluation II

BMI – body mass index

BZD – benzodiazepines

CLP – cecal ligation and puncture

CRP – C-reactive protein

DBI – diazepam-binding inhibitor

GABA – gamma-aminobutyric acid

GABA_A_ R – gamma-aminobutyric acid type A receptor

ICU – intensive care unit

IL – interleukin

LPS – lipopolysaccharide

mAb – monoclonal antibody

PICS – post-intensive-care syndrome

PSS – post-sepsis syndrome

PTSD – post-traumatic stress disorder

RCT – randomized controlled trial

ROC – receiver operating characteristic

SAPS II – simplified acute physiology score II

SASP – senescence-associated secretory phenotype

SIRS – systemic inflammatory response syndrome

SOFA – sequential organ failure assessment

p21 – cyclin-dependent kinase inhibitor 1A (CDKN1A) protein
